# The immunotoxin targeting PRLR increases tamoxifen sensitivity and enhances the efficacy of chemotherapy in breast cancer

**DOI:** 10.1186/s13046-024-03099-4

**Published:** 2024-06-20

**Authors:** Jiawei Zhang, Junjun Liu, Yali Yue, Lei Wang, Qunye He, Shuyi Xu, Junyan Li, Yunji Liao, Yu Chen, Shusheng Wang, Yueqing Xie, Baohong Zhang, Yanlin Bian, Dimiter S. Dimitrov, Yunsheng Yuan, Jianwei Zhu

**Affiliations:** 1 Engineering Research Center of Cell & Therapeutic Antibody, MOE, School of Pharmacy, Shanghai Jiao Tong University, Building 6, Room 208, 800 Dongchuan road, Shanghai, 200240 China; 2 Jecho Laboratories, Inc, Frederick, MD 21704 USA; 3 Jecho Biopharmaceuticals Co., Ltd, Tianjin, 300467 China; 4 University of Pittsburgh Department of Medicine, Pittsburgh, PA 15261 USA

## Abstract

**Background:**

Though tamoxifen achieves success in treating estrogen receptor α (ERα)-positive breast cancer, the followed development of tamoxifen resistance is a common challenge in clinic. Signals downstream of prolactin receptor (PRLR) could synergize with ERα in breast cancer progression. However, the potential effect of targeting PRL-PRLR axis combined with tamoxifen has not been thoroughly investigated.

**Methods:**

High-throughput RNA-seq data obtained from TCGA, Metabric and GEO datasets were analyzed to explore PRLR expression in breast cancer cell and the association of PRLR expression with tamoxifen treatment. Exogenous or PRL overexpression cell models were employed to investigate the role of activated PRLR pathway in mediating tamoxifen insensitivity. Immunotoxin targeting PRLR (N8-PE24) was constructed with splicing-intein technique, and the efficacy of N8-PE24 against breast cancer was evaluated using in vitro and in vivo methods, including analysis of cells growth or apoptosis, 3D spheroids culture, and animal xenografts.

**Results:**

PRLR pathway activated by PRL could significantly decrease sensitivity of ERα-positive breast cancer cells to tamoxifen. Tamoxifen treatment upregulated transcription of PRLR and could induce significant accumulation of PRLR protein in breast cancer cells by alkalizing lysosomes. Meanwhile, tamoxifen-resistant MCF7 achieved by long-term tamoxifen pressure exhibited both upregulated transcription and protein level of PRLR. Immunotoxin N8-PE24 enhanced sensitivity of breast cancer cells to tamoxifen both in vitro and in vivo. In xenograft models, N8-PE24 significantly enhanced the efficacy of tamoxifen and paclitaxel when treating PRLR-positive triple-negative breast cancer.

**Conclusions:**

PRL-PRLR axis potentially associates with tamoxifen insensitivity in ERα-positive breast cancer cells. N8-PE24 could inhibit cell growth of the breast cancers and promote drug sensitivity of PRLR-positive breast cancer cells to tamoxifen and paclitaxel. Our study provides a new perspective for targeting PRLR to treat breast cancer.

**Supplementary Information:**

The online version contains supplementary material available at 10.1186/s13046-024-03099-4.

## Highlights

Tamoxifen up-regulates PRLR protein level in breast cancer cells and activation of PRLR pathway by PRL could decrease drug-sensitivity of breast cancer cells to tamoxifen.

The immunotoxin targeting PRLR could reverse drug-sensitivity to tamoxifen in tamoxifen-resistant breast cancer in vitro and in vivo.

The immunotoxin targeting PRLR significantly improve the efficacy of chemotherapy in PRLR-positive TNBC and xenograft models.

**Supplementary Information:**

The online version contains supplementary material available at 10.1186/s13046-024-03099-4.

## Introduction

Prolactin, predominantly secreted by lactotrophs within the anterior pituitary gland, exerts its physiological role primarily in the lactating mammary gland [1]. However, emerging evidence suggests potential involvement of PRL in breast cancer (BC) pathogenesis, particularly in its capacity to promote tumor growth. Notably, clinical studies have identified PRL as a potential risk factor for ERα-positive BC [2, 3]. Prolactin receptor (PRLR), which is the binding receptor for PRL, has been suggested to be upregulated in hormone receptor (HR)-positive BC tissues, further indicating a link between PRL signaling and BC progression [4, 5].

Studies demonstrate that PRL binds to PRLR and promotes BC cells proliferation by activating multiple downstream signal pathways, such as ERK1/2, STAT3/5, Src family and PI3K/AKT [6–11]. Moreover, PRL could activate ERα by phosphorylating AF-1 domain at Ser118/167, a process that is facilitated by PI3K/AKT or MEK/ERK pathways, and could induce ERα-positive BC [12–16]. Physiologically, activation of dopamine receptor could suppress PRL transcription in lactotrophs through regulating Pit-1 promoter [17]. However, dopamine receptor agonists, such as cabergoline and bromocriptine, have not yielded the expected clinical benefits [18–21]. Thus, studies have been conducted to further explore whether the autocrine PRL expressed by cancer cells could contribute to cancer progression. Indeed, studies in mouse models and clinical investigations have demonstrated that autocrine PRL derived from tumor cells could induce and promote BC [12, 13, 22–24]. Therefore, targeting the autocrine PRL becomes imperative to better understand PRL’s role in BC. LFA102, a monoclonal antibody (mAb) that targets PRLR, has demonstrated efficacy in antagonizing PRL-induced signals [25]. However, despite its potential antagonistic properties against PRLR, LFA102 has not shown persuasive benefits in clinical trials, indicating a single-targeted approach to PRLR is insufficient to suppress clinical cancer progression [26, 27]. Likewise, G129R, a PRL mimics that competes with PRL for binding PRLR, effectively antagonizes PRL but demonstrates limited anti-tumor effects [28–30].

PRL-PRLR pathway plays a complicated role in regulation of ERα-positive BC progress and engages in the crosstalk with multiple crucial factors, such as estrogen, epidermal growth factor (EGF) and insulin-like growth factor-I [31, 32]. Over 70% of BCs in women expressed ERα, and endocrine therapies were conventional treatment for ERα-positive BC [33, 34]. In BC cells, estradiol can promote PRLR transcription through activating ERα [35]. Besides, estradiol could stimulate PRL transcription by enhancing AP-1 and ERE activity in BC, thereby amplifying the pro-tumor potential of autocrine or paracrine PRL within the tumor microenvironment [36]. Conversely, PRL could activate ERα independent of estradiol but dependent of ERK [14]. Additionally, through phosphorylating PAK1, PRL could lead to ERα phosphorylation at S305, which subsequently leads to the transactivation of ERα by phosphorylating S118 [16]. In addition to enhancing unliganded ERα signaling, PRL treatment also increases ERα expression [37]. Collectively, ERα and PRL cooperatively promote downstream pro-tumor signals and BC proliferation [38, 39]. Prior study has shown that blocking simultaneously ERα and PRLR pathway could effectively inhibit breast tumor growth in animal models [25]. Tamoxifen, a clinical standard selective estrogen modulator, is widely applied for premenopausal women according to NCCN guidelines. However, approximately 40% of patients treated with tamoxifen would eventually develop resistance to tamoxifen [40]. The ERK pathway, which plays a crucial role in both the ERα and PRL-PRLR pathways, has been implicated to be involved in the development of tamoxifen resistance in BC [41, 42]. It’s well known that drug resistance is one of the biggest challenges in the cancer therapy. Based on the crosstalk between the ERα and PRLR pathways, targeting PRLR therapy might offer a promising strategy for tamoxifen-resistant BC. Several PRLR-targeting agents has been developed. For instance, PRLR×CD3 bispecific antibody efficiently activates T cells to inhibit cancer cells [43]. ABBV-176, a PRLR targeting antibody-drug conjugate (ADC), significantly suppresses cancer progression [44]. However, ABBV-176 has been associated with cumulative toxicity in a phase I clinical trial [45]. Immunotoxins, which are chimeric molecules composed of a protein toxin fused to a targeting moiety, offer advantages over ADCs by demonstrating reduced payload disassociation and off-target toxicity [46–48]. In our study, we constructed PRLR-targeting immunotoxin and combined it with tamoxifen to treat multiple BC cell lines, including tamoxifen-resistant MCF7 cells. The drug combination exhibited persuasive effect both in vitro and in vivo. Intriguingly, tamoxifen upregulated PRLR protein expression in BC cells, which further provided pharmacological rationale for the drug combination.

## Materials and methods

### Cell culture and transfection

T47D cells were maintained in RPMI-1640 (Gibco, USA) supplemented with 10% FBS (Hyclone, USA). MCF7 and MDA-MB-231 cells were maintained in DMEM (Gibco) supplemented with 10% FBS. MCF7-TAMR cells were acquired by culturing MCF7 with 1–5 µM tamoxifen (HY-13,757 A, MCE, USA) for 6 months. T47D-TETON-PRL, MCF7-TETON-PRL, MCF7-TAMR-TETON-PRL and MDA-MB-231-PRLR cells were acquired by infecting cells with pLVX-Puro lentivirus packaged in biosafety level-2 laboratory.

### Acquisition and bioinformatic analysis of cell line and clinical datasets

TCGA BC data was downloaded from Xena (https://xenabrowser.net/datapages/) [49]. METABRIC data was downloaded from cbioportal (http://www.cbioportal.org/) [50–52]. Data of relapse-free survival was acquired and analyzed on KM-plotter (https://kmplot.com/analysis/) [53]. GSE67916, GSE125738, and GSE147271 datasets were downloaded from GEO database (https://www.ncbi.nlm.nih.gov/geo/) [54, 55]. All the data was analyzed with R (version 4.1.3).

### Western blot

After treatment, cells were washed for three times with cool PBS and then lysed with cell lysis buffer for Western blotting and immune-precipitation (IP) (P0013, Beyotime, China). 10–30 µg protein were separated on 6-12% SDS-PAGE and then were transferred by electrophoresis to PVDF membrane (IPVH00010, Merck, German). Membranes were then blocked for 1 h under room temperature in 5% BSA (ST023, Beyotime, China) solved in TBST. Subsequently, membranes were incubated with primary and secondary antibodies. The proteins were detected by NcmECL reagent (P10300, NCM Biotech, China). Antibodies targeting p-ERα-S118 (ab32396, Abcam, England), ERα (ab108398, Abcam, England), p-ERK1/2-T202/T204 (4377, CST, USA), ERK1/2 (4695, CST, USA), β-actin (4970, CST, USA), STAT3 (ab68153, Abcam, England), p-STAT3-Y705 (ab76315, Abcam, England), STAT5 (ab16276, Abcam, England), p-STAT5-Y694 (ab32364, Abcam, England), PRLR (ab170935, Abcam, England), rabbit IgG-HRP-linked (7074), mouse IgG-HRP-linked (7076) were used.

### q-PCR

The RNA was extracted from cells using Ultrapure RNA Kit (CW0581, CWBIO, China). Then cDNA was prepared by RNA reverse transcript with PrimeScript RT Master Mix (RR036, Takara, Japan). qPCR was conducted following the instruction of TB Green Premix Ex Taq (RR420, Takara, Japan). Primers in this study: β-actin-F: 5’ CACCATTGGCAATGAGCGGTTC 3’, β-actin-R: 5’ AGGTCTTTGCGGATGTCCACGT 3’, PRLR-F: 5’ CATGGTGACCTGCATCTTTCCG 3’, PRLR-R: 5’ GTGGGAGGAAAGTCTTGGCATC 3’.

### Cell viability assay

Two thousand cells were seeded in 96-well plate one day before treatment. Respective reagents were added to the cells after attached to the wells. After 48–120 h, 10% CCK8 reagent was added to the cells according to instructions. The plate was left under 37℃ for 1–3 h. Then absorbance at 450 nm were measured with microplate reader (Infinite M200 pro, Tecan, Swiss). For experiments with prolactin, cells were first cultured in completed phenol red-free medium supplemented with charcoal-stripped serum (12676-029, Gibco, USA) for three days to exclude the influence of other hormones.

### Cell apoptosis assay

Fifty thousand cells were seeded in 6-well plate one day before treatment. After cells were attached, 20 µg/ml N8-PE24 were added to the cells. 48 h later, cells were digested and collected by trypsin without EDTA. The apoptosis of cells was detected under the instructions of Annexin V-FITC/PI kit (70-AP101-100, Multisciences Biotech, China).

### Lysosome pH measurement

Cells were plated into 96-well flat bottom plate one day before measurement. Before measurement, agents of interest were added to the wells cultured for 10 min. Then, 2µM PDMPD (40768ES50, Yeasen, China) was added to each well and cells were cultured at 37℃ for 10 min. The Em440/540 excited by Ex329/384 was determined by plate reader. Em440/540 was converted to pH by calibration with KCl buffered to pH 3.5 to 5.5 in the presence of 10 µM monensin and 20 µM nigericin.

### Antibody screening

Phage library display (Jecho Laboratories Inc. USA) was conducted to screen PRLR-targeting antibody. Briefly, a scFv phage library was exposed to coated PRLR, after which the unbound phages were washed away. The left phage was cultured and expanded. Several rounds of screening were conducted to enrich targeted phages. Last, genes of phages were cloned for antibody construction. ELISA was used to identify the binding capacity of antibodies screened.

### ELISA

For testing the binding activity of anti-PRLR mAbs, two hundred nano grams of PRLR-HIS recombinant protein (PRP-H5251, ACROBiosystems, USA) dissolved in 50mM carbonate buffer (pH 9.6) was coated on 96-well ELISA plate under 4℃ overnight and then the wells were washed three times with PBST. After blocking the wells with 5% BSA-PBST at 37℃ for 1 h, the wells were cultured with serial diluted antibodies at 37℃ for 1 h. After washing the wells with PBST for three times, 1:10000 anti-human IgG-HRP-linked antibody (32,935, CST, USA) dissolved in 5% BSA-PBST was added to each well and the plate was cultured at 37^o^C for 1 h. Last, the wells were washed for three times and the absorbance at 450 nm was measured by a microplate reader (Infinite M200 Pro, Tecan, Swiss). For quantifying the concentration of PRL in cell culture medium, human PRL ELISA kit (D711066, Sangon Biotech, China) was used. The experiment was done according to the instruction. First, the cell culture medium was collected and passed through 0.22 μm filter.

Then, the culture medium and serial diluted standard PRL solution were added to the ELISA plate, which was provided by the kit and had already been coated with anti-PRL first antibody. Following that, the plate was cultured under 37℃ for 90 min. After washing the plate for three times, biotin-labeled anti-PRL secondary antibody was added to each well and the plate was left under 37℃ for 60 min. Then, the wells were washed for three times before HRP-labeled anti-biotin antibody was added. The plate was cultured under 37℃ for 30 min. Last, after washing the wells, TMB substrate solution provided by the kit was added to the wells. After 20 min, the reaction was stopped by stop solution provided by the kit. Absorbance at 450 nm was measured by microplate reader.

### Flow cytometry

Twenty thousand cells were resuspended in pre-cool FACS buffer (2% FBS-PBS). Then 1:200 anti-PRLR-APC antibody (10,278-R204-A, Sino Biological, China) was added to the cells. Cells were kept in dark place on ice for 30 min. Then cells were washed for three times with pre-cool FACS buffer and resuspended in 200µL FACS for analyzed on flow cytometer (Cytoflex, Beckman, USA).

### 3D spheroid culture

Cells were collected and resuspended at 2 × 10^4^ cells/mL in medium containing 5% FBS. For T47D and MCF7, 10nM estradiol should be added to the medium. Then 100µL of cells was added to each well of 96-well ultra-low attachment plate (7007, Corning, USA) and was centrifuged at 300 g for 10 min. After 72-hours cultural under 37℃, indicated drugs were added to the wells. Subsequently, the spheroids were cultured for 10 days to observe the effect of the drugs. Celltiter Glo was exploited to quantify the viability of spheroids.

### Cycloheximide degradation assay

Twenty thousand cells were seeded in 12-well plate for the experiment. Bafilomycin (HY-100,558, MCE, USA), MG132 (HY-13,259, MCE, USA) or tamoxifen was added to the cells 1 h before cycloheximide treatment. Then cycloheximide (14126-1, Cayman Chemical, USA) was added to the cells for indicated time. Cells were lysed at different time points for Western blotting. MCF7-PRLR-EGFP cells were fixed by 4% paraformaldehyde and analyzed with fluorescence microscopy.

### Split intein platform to construct N8-PE24 immunotoxin

The split intein platform was described previously in detail [56, 57]. First, Int-N fragment is linked behind N8-Fab and PE24 is linked behind Int-C fragment. N8-Fab-Int-N was expressed by 293F system and Int-C-PE24 was expressed by an *E.coli* system. Then the two fragments were mixed at ratio of 1:2 in PBS, pH8.0. 100µM of DTT was added to the mixture for switching on split intein reaction in a 37℃ water bath for 4 h. Subsequently, the mixture was dialyzed to PBS, pH 8.0 to thoroughly remove DTT. Then 8mM oxidized glutathione was added to the mixture that was kept in 4℃ for at least 24 h to complete the reaction. Last, N8-PE24 was purified by Q-sepharose column.

### Internalization assay

The internalization of an antibody or N8-PE24 was measured by a flowcytometry and a fluorescence microscope. For flowcytometry, 20 µg antibody or N8-PE24 was first cultured with T47D cells on ice for 30 min. Then the unbound antibody or N8-PE24 was washed away. Cells were kept at 37℃ for internalization. Antibody or N8-PE24 bound on cell membranes was detected by the anti-human IgG antibody with a flowcytometry. For fluorescence, pHrodo-red (P36600, Thermo Fisher Scientific, USA) was conjugated to N8-PE24 first. Then N8-PE24-red was added to cells and cultured at 37℃ for 4–8 h. The fluorescence was observed on fluorescence microscope.

### Animal experiment

All animal experiments were conducted in compliance with guidelines from Institutional Animal Care and Use Committee (IACUC) of Shanghai Jiao Tong University. For MCF7-TAMR experiment, SPF-grade NOD/SCID female mice, aged 5 weeks, were first embedded with 17β-estradiol pellets (SE-121, innovrsrch, USA) subcutaneously one week before cells implantation. One day before the inoculation, cell culture medium of MCF7-TAMR was changed for fresh DMEM supplemented with 10% FBS. On the day of inoculation, cells were first washed with cool PBS. Then the cells were digested by trypsin and resuspended in pre-cooled DMEM. After that, cells were counted and diluted to a final concentration of 2 × 10^8^ cells/ml. Then equal volume of Matrigel (356234, Corning, USA) kept on ice was mixed with cells. Finally, 1 × 10^7^ MCF7-TAMR cells (100µL) were injected subcutaneously on the armpit of mice. For MDA-MB-231-PRLR experiment, SPF grade nude female mice, aged 6 weeks, were injected subcutaneously on the armpit with 5 × 10^6^ cells. The protocol for generating MDA-MB-231 xenograft was similar to MCF7-TAMR, except for that MDA-MB-231 did not need 17β-estradiol pellets. When tumor reached a volume of 100mm^3^, the treatment began.

### Immunohistochemistry

Breast cancer microarrays (F151Br01 and F551101, Bioaitech, China) and BC tissues from xenografts were used for IHC staining. For immunohistochemistry, tumors were fixed in 4% paraformaldehyde (Freethinking, Nanjing, China) for 24 h, and embedded in paraffin. Tumor sections were cut, followed by deparaffinization, heat antigen retrieval and endogenous peroxidase blocking. Then, the tumor sections or microarrays were blocked with 3% bovine serum albumin in PBS for 30 min and incubated with anti-human PRLR rabbit antibody (ab170935, Abcam, England) or anti-ki67 rabbit antibody (ab15580, Abcam, England) at 4 °C for overnight. HRP-labeled goat anti-rabbit IgG (Freethinking, Nanjing, China) were then added and incubated for 50 min. Detection was conducted with DAB detection kit (Dako, Carpinteria, CA, USA) according to manufacturer’s instructions. The tumor sections were counterstained with hematoxylin (Freethinking, Nanjing, China). Images were acquired using the OLYMPUS BX53 Microscope.

### Statistical analysis

Graphpad (version 8.0.2) was used for visualizing the data. Data are shown as mean ± standard deviation (SD) and statistical analysis was based on two-tailed heteroscedastic Student’s t-test or One-way ANOVA. Statistical significance was set at *p* < 0.05. p > = 0.05 (NS), *p < 0.05, **p < 0.01, ***p < 0.001, ****p < 0.0001.

## Results

### PRLR was associated to ERα expression and tamoxifen treatment in the breast cancer cells

**Fig. 1:**
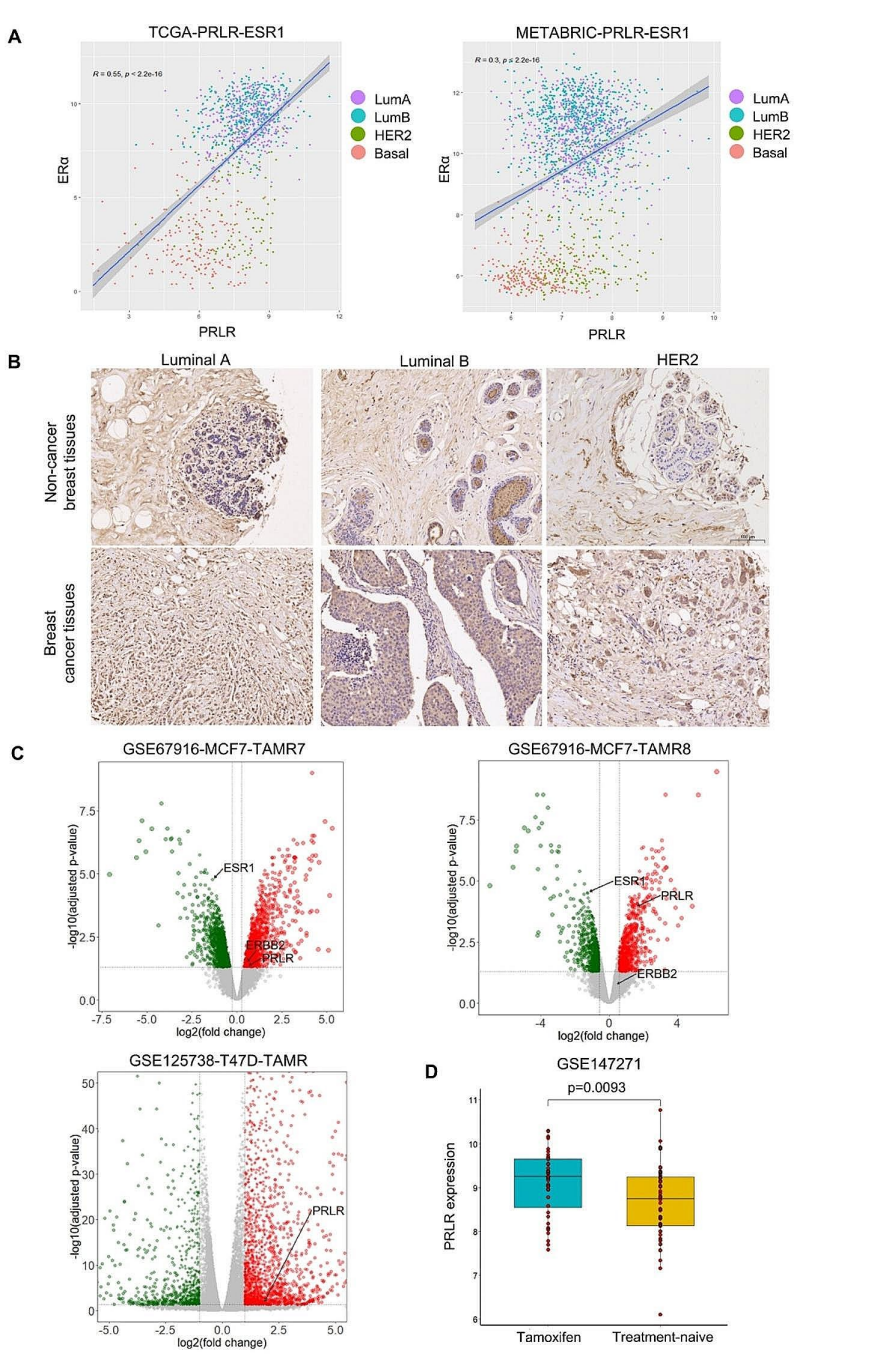
PRLR transcription shared similar pattern with ERα and was upregulated in multiple tamoxifen-resistant breast cancer cells. (**A**) Linear curve fitted between PRLR and ERα (ESR1) level based on TCGA and METABRIC database. (**B**) Immunohistochemical staining of PRLR on breast cancer tissue of different types. (**C**) Volcano plot of RNA sequencing results based on GEO dataset. MCF7-TAMR datasets were sourced from GSE67916 and T47D-TAMR dataset was from GSE125736. (**D**) PRLR level of tamoxifen treated (post tamoxifen treatment, blue) and treatment-naïve (prior to tamoxifen treatment, yellow) breast cancer tissues. The dataset was sourced from GSE147271

PRL-PRLR pathway plays a complicated role on the progression of ERα-positive BC and engages in intricate crosstalk with various crucial factors [31, 32]. The interplay between PRLR and ERα has been identified as a pivotal axis within hormone receptor-positive BC cells. However, the impact of tamoxifen treatment on PRLR level has not been fully explored. To understand whether tamoxifen treatment could potentially affect PRLR level, we initiated our investigation by analyzing high-throughput RNA-seq data sourced from TCGA, METABRIC and GEO database. It was widely reported that PRLR expression correlated with ERα level across BC cell lines [4, 14]. In consistent with the reports, RNA sequencing data from The Cancer Genome Atlas (TCGA) and METABRIC revealed a positive correlation between PRLR and ERα expression levels (Fig. 1A and Figure S1A). Based on our initial findings, we further identified that luminal A BC tissues exhibited a higher expression of PRLR compared to their normal tissue counterparts, as evidenced by our examination of a tissue chip (Fig. 1B and FigureS1B-C). Additionally, PRLR level could be upregulated when tamoxifen resistance occurred, as exemplified in MCF7-TAMR7/8 and T47D-TAMR cell lines, which exhibited significantly increased PRLR expression relative to their respective controls (Fig. 1C). In a clinical analysis, tissues from BC patients who underwent tamoxifen treatment exhibited a significant higher expression of PRLR compared to those from patients who had not received tamoxifen therapy (Fig. 1D). In summary, these findings identified the paralleled expression pattern between PRLR and ERα in breast cancer. Besides, tamoxifen treatment could potentially further promote PRLR expression.

### Activation of PRLR pathway desensitized breast cancer cells to tamoxifen treatment

**Fig. 2:**
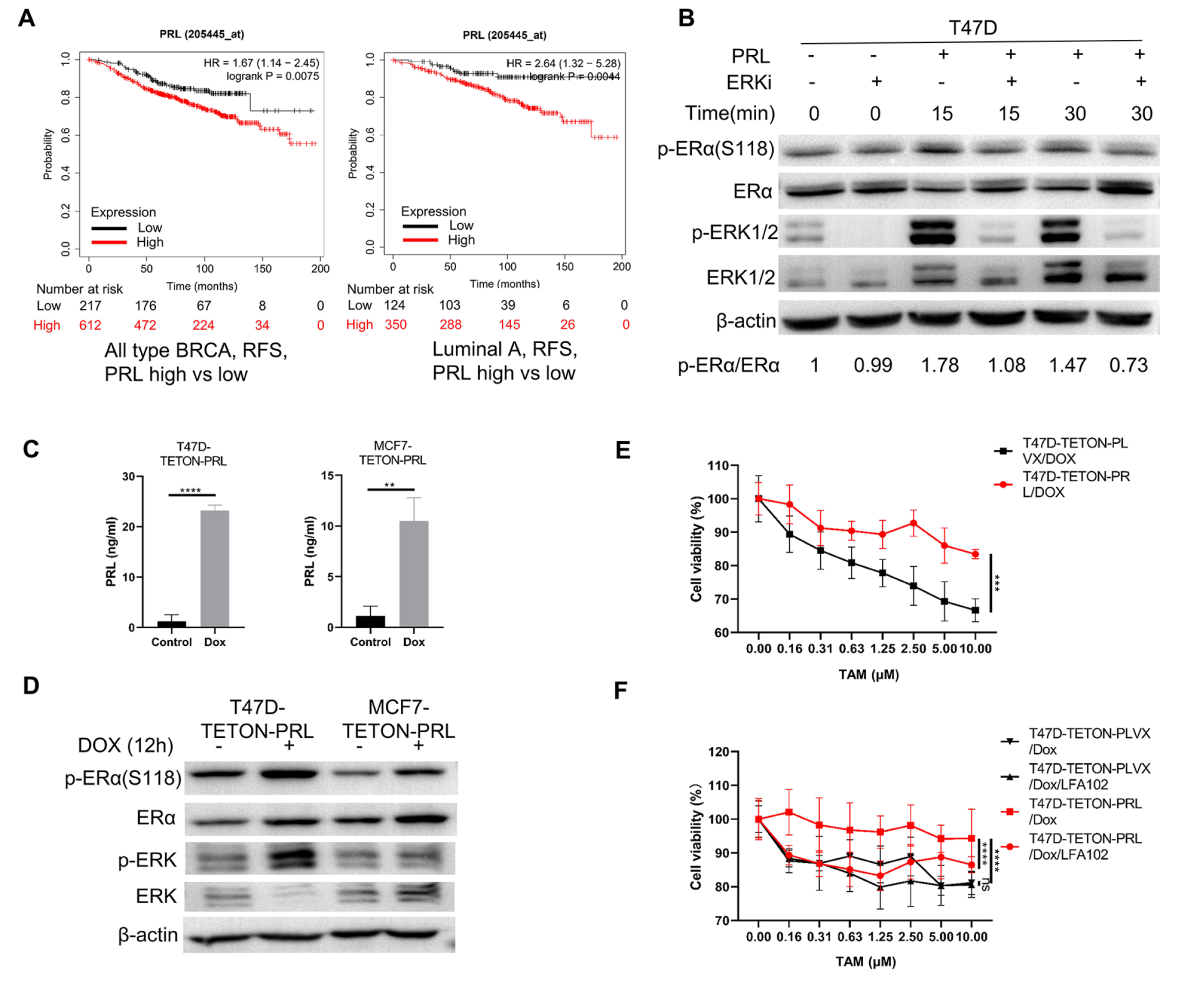
PRL induced tamoxifen resistance in breast cancer cells. (**A**) Comparison of RFS between patients of PRL-high and PRL-low breast cancer tissues. All the patients were treated with tamoxifen. Left: RFS analysis of all types of BRCA. Right: RFS analysis of Luminal A type BRCA. (**B**) Western blot detecting phosphorylated ERα on S118 (functional site located in AF-1 domain) and ERK on T202/204 activated by PRL in the presence or absence of ERK inhibitor SCH772984. Standardized quantification of p-ERα against ERα was performed. Cells were starved in DMEM without FBS for 24 h prior to activation of PRL. (**C**) ELISA detecting PRL level in cell culture medium when Tet-on PRL was induced by doxycycline for 12 h. (**D**) Western blot detecting phosphorylated ERα and ERK when Tet-on PRL was induced by doxycycline for 12 h. (**E**) Evaluation of T47D viability in the presence of tamoxifen when PRL was induced by doxycycline. For each group, viability of cells without tamoxifen treatment was set as 100%. (**F**) Evaluation of T47D viability in the presence of tamoxifen when PRL was induced in the presence of LFA102 mAb or not. For each group, viability of cells without tamoxifen treatment was set as 100%

Previous study has demonstrated that the activation of PRLR pathway could regulate phosphorylation of ERα via ERK pathway, a process that was independent of estradiol [14]. Given that PRL served as the ligand for PRLR, it was speculated that PRL could influence the response to tamoxifen by activating PRLR pathway. To identify the potential effect of PRL on the tamoxifen treatment, we analyzed the prognosis data from a cohort of BC patients who received tamoxifen treatment. Consistent with our expectation, high PRL level in BC tissues was associated with shorter relapse-free survival (RFS) in patients undergoing tamoxifen treatment (Fig. 2A). It indicated that local prolactin produced by breast cancer cells could affect the response and prognosis of tamoxifen treatment. To investigate the potential effect of prolactin on tamoxifen response, we initially demonstrated that PRL could regulate phosphorylation of ERα at Ser118, a process that could be abrogated by SCH772984, a selective ERK inhibitor (Fig. 2B). Subsequently, we generated T47D-TETON-PRL and MCF7-TETON-PRL cell lines that could express prolactin upon doxycycline induction (Fig. 2C and Figure S2A). Upon successful induction of PRL by doxycycline, we observed that the overexpression of PRL, leading to PRLR pathway activation, could also trigger phosphorylation of both ERK and ERα in T47D-TETON-PRL and MCF7-TETON-PRL cell lines (Fig. 2D). Furthermore, activation of PRLR pathway by overexpression of PRL conferred resistance to tamoxifen in T47D cells, an effect that was efficiently neutralized by LFA102, an antibody that antagonizes PRLR (Fig. 2E-F). A similar, albeit less pronounced, effect of prolactin was observed in MCF7 cells, which could potentially be attributable to their lower PRLR expression compared with T47D (Figure S2B-C). Collectively, these results suggested that PRL could regulate phosphorylation of ERα independent of estradiol and consequently reduce cell sensitivity of BC to tamoxifen.

### Tamoxifen upregulated PRLR level in breast cancer cells

**Fig. 3:**
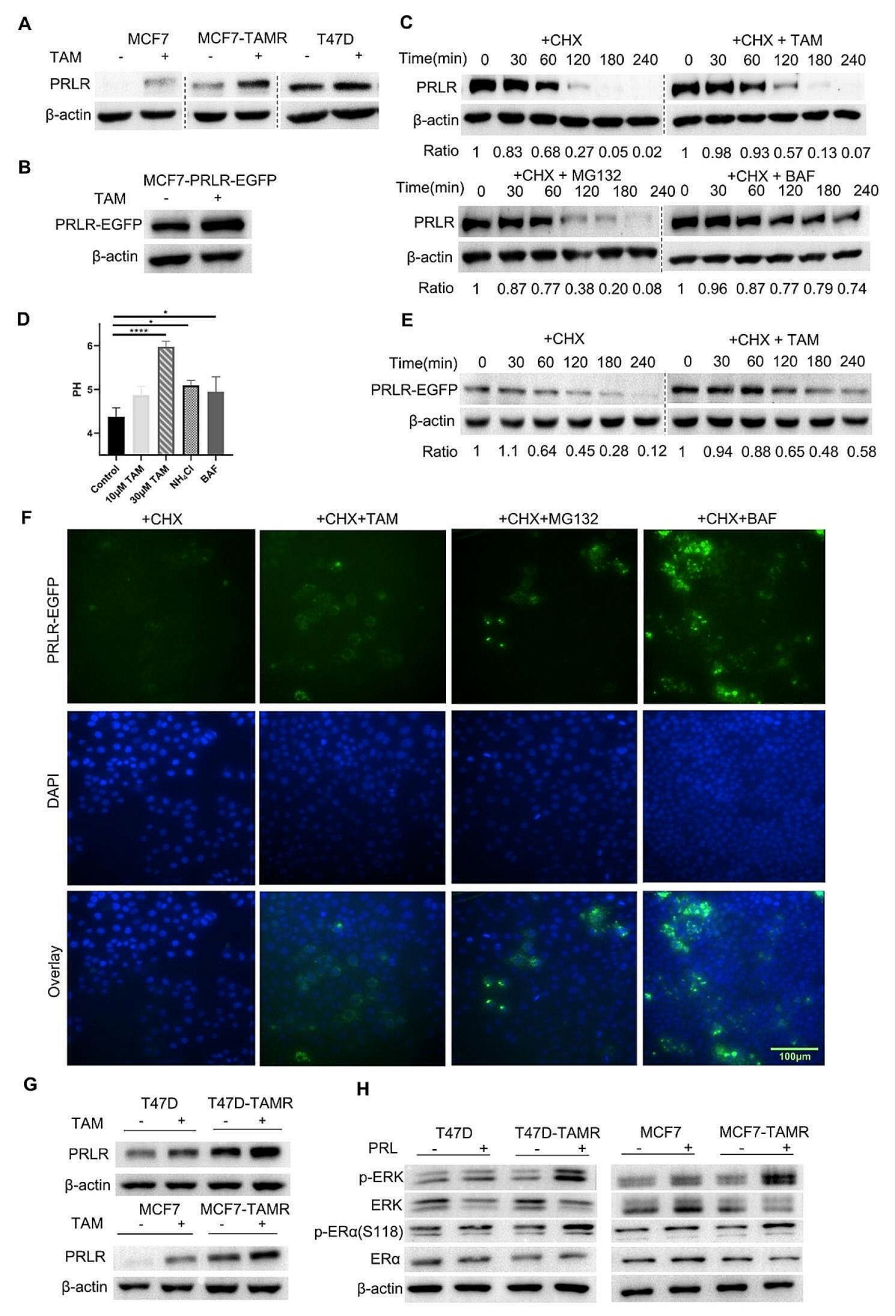
Tamoxifen alkalized lysosome and upregulated PRLR protein level. (**A**) Western blot detecting PRLR protein level in MCF7-TAMR, original MCF7 and T47D cells with or without tamoxifen treatment. (**B**) Western blot detecting PRLR protein level in MCF7 cells expressing exogenous PRLR-EGFP when tamoxifen was present or not. MCF7-PRLR-EGFP was generated by infecting MCF7 with PRLR-EGFP lentivirus. Cells were lysed and PRLR was probed. (**C**) Cycloheximide chase assay to determine PRLR degradation in T47D cells when tamoxifen was present or not. Cycloheximide was added to the cells 15 min before treatment of indicated reagents. At indicated time, cells were lysed and PRLR was probed. MG132: a proteasome inhibitor. BAF: Bafilomycin, a lysosome inhibitor. (**D**) PDMPO lysosome probe was exploited to determine lysosome pH in MCF7-TAMR cells in the presence of tamoxifen, NH_4_Cl or bafilomycin. Em440/540 was recorded and converted to pH value according to standard Em440/540-pH curve. (**E**) Cycloheximide chase assay to determine exogenous PRLR-EGFP degradation in MCF7-PRLR-EGFP cells when tamoxifen was present or not. At indicated time, cells were lysed and PRLR was probed. (**F**) Immunofluorescence of PRLR-EGFP (green) and nucleus (blue) in MCF7-PRLR-EGFP upon treatment of cycloheximide and indicated reagents for 2 h. (**G**) Western blot analysis showed upregulated PRLR level in T47D-TAMR and MCF7-TAMR cells compared to original T47D and MCF7 cells. (**H**) Western blot detecting p-ERK, p-ERα(S118) activated by PRL in T47D-TAMR and MCF7-TAMR cells. Cells were starved in DMEM devoid of FBS for 24 h prior to activation of PRL for 15 min

PRL and estradiol could reciprocally regulate the expression level of each other’s receptor [14, 35]. However, elucidating the effect of tamoxifen on PRLR level is of significant interest. Thus, we treated multiple BC cell lines with tamoxifen to explore the connection between tamoxifen and PRLR. Results from the Western blots demonstrated that exposure to tamoxifen significantly increased the protein level of PRLR in T47D, MCF7, and tamoxifen-resistant MCF7 cells (MCF7-TAMR), and it significantly upregulated the transcription level of PRLR (Fig. 3A and Figure S3A). Subsequently, we generated a stable MCF7 cell lines overexpressing PRLR-EGFP (MCF7-PRLR-EGFP) to investigate whether tamoxifen could affect exogenous PRLR level. Notably, tamoxifen also promoted the exogenous PRLR level in MCF7-PRLR-EGFP cells, prompting us to investigate the degradation pathway of PRLR (Fig. 3B). Physiologically, PRLR was known to be constitutively trafficked to lysosomes, where PRLR underwent rapid degradation [58, 59]. In our study, with CHX chase assay, we identified that lysosome inhibitor bafilomycin A1 (BAF) could significantly retard the degradation process of PRLR.(Fig. 3C). Notably, the degradation of PRLR could also be impeded by tamoxifen (Fig. 3C). Although the proteasome MG132 was also observed to modestly extend half-life of PRLR protein, the ubiquitination status of PRLR remained unaltered by tamoxifen, suggesting that the increases in PRLR protein level induced by tamoxifen was predominantly associated with lysosomal degradation pathway (Fig. 3C and Figure S3C). Tamoxifen, except for being a selective estrogen receptor modulator, was also known for its ability to increase the pH within lysosomes, a process that might subsequently induce lysosome damage in BC cells [60–62]. Correspondingly, we confirmed the alkalizing property of tamoxifen with a lysosome pH test (Fig. 3D and Figure S3B). We further identified tamoxifen’s effect in hindering the degradation of exogenous PRLR within MCF7-PRLR-EGFP cells (Fig. 3E-F). These findings collectively suggested that short-term tamoxifen treatment could directly affect PRLR protein levels, potentially through the inhibition of lysosomes. As previously reported, PRLR was upregulated in several tamoxifen-resistant BC lines including MCF7-TAMR7/8 (Fig. 1C). Considering the slight but significant upregulation of PRLR mRNA caused by short-term tamoxifen exposure, we wondered if long-term tamoxifen pressure could further promote PRLR level. To test it, we screened tamoxifen-resistant T47D-TAMR and MCF7-TAMR cell lines by continuously culturing T47D and MCF7 with increasing doses of tamoxifen, which was followed by identifying their reduced sensitivity to tamoxifen Figure S4A). Subsequent analysis showed both upregulated PRLR transcription and protein level in T47D-TAMR and MCF7-TAMR cells (Fig. 3G and Figure S4B). Furthermore, T47D-TAMR and MCF7-TAMR cells demonstrated heightened sensitivity to prolactin, as evidenced by higher p-ERK and p-ERα level in response to PRL (Fig. 3H). In summary, these results collectively suggested that PRLR level could be upregulated after tamoxifen treatment.

### Monoclonal anti-PRLR antibody N8 inhibited PRLR pathway and promoted internalization into the breast cancer cells

**Fig. 4:**
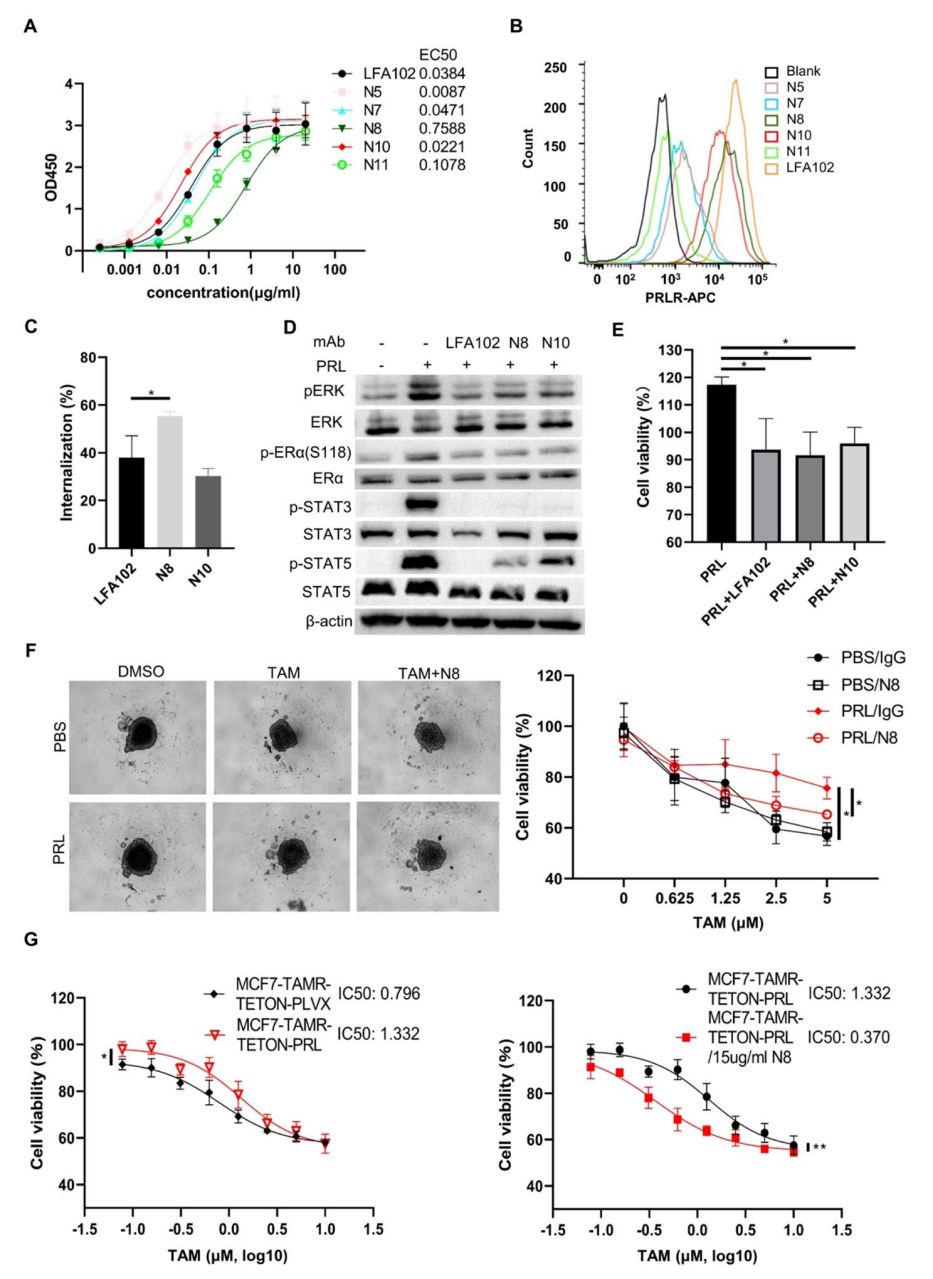
Analyzing bioactivity of monoclonal anti-PRLR antibodies. (**A**) Binding of mAbs on recombinant PRLR was determined by ELISA. The ELISA plate was pre-coated with recombinant PRLR protein. Serial diluted mAbs were added to the wells as primary antibodies. (**B**) Binding of mAbs on T47D was determined by flowcytometry. Indicated mAbs were added to the cells as primary antibodies. After that, antibodies bound on cell membranes were detected by anti-human Fc-APC antibody. (**C**) Flowcytometry was used to determine internalization of PRLR-targeting mAbs. The cells were cultured with indicated antibody on ice for 60 min to saturate the cell membranes with antibody. Subsequently, cells were transferred to 37℃ to start the internalization. Antibodies left on cell membranes under 37℃ were detected by anti-human-Fc-APC at 0 h and 1 h. Internalization (%) was calculated by [MFI (0 h) – MFI (1 h)]/MFI (0 h). (**D**) Western blot detecting p-ERK (T202/T204), ERK, p-ERα (Ser118), ERα, p-STAT3 (Y705), STAT3, p-STAT5 (Y694), STAT5 and β-actin (loading control) in T47D cells stimulated by PRL in the presence of indicated PRLR-targeting mAbs. Cells were starved in DMEM devoid of FBS for 24 h prior to activation of PRL for 15 min. (**E**) Cell viability of T47D cells was determined by CCK8 in the presence of PRL with indicated PRLR-targeting mAbs. Cells were cultured for 72 h before viability was tested. Viability of cells without any treatment was set as 100%. (**F**) Viability of T47D spheroid was determined by Celltiter-glo in the presence of PRL or N8 mAb. Left: the image of spheroids in indicated groups. Right: Curve of the viability of T47D spheroids to tamoxifen concentration. Viability of T47D spheroid treated without any treatment (PBS/IgG/0µM tamoxifen) was set as 100%. (**G**) Cell viability of MCF7-TAMR cells was determined when overexpression of PRL was induced (Left) or N8 mAb was present (Right). Cell viability of MCF7-TAMR cells without any treatment (0µM tamoxifen/0µg/ml N8) was set as 100%

Identifying PRLR as a potential therapeutic target, we comprehensively screened several antibodies for targeting PRLR. We conducted ELISA to confirm that all the antibodies could efficiently bind to the plate-coated recombinant PRLR protein (Fig. 4A). Consistently, all the antibodies exhibited robust binding capacity as measured by Surface Plasmon Resonance (SPR) assay (Surface Plasmon Resonance) (Figure S5A). Subsequently, we performed flowcytometry and found that both antibodies N8 and N10 were particularly efficient at binding to the naturally presented PRLR on T47D cells (Fig. 4B). Notably, antibody N8 distinguished itself by demonstrating strong internalization capabilities (Fig. 4C). Furthermore, N8 efficiently inhibited PRL-induced phosphorylation of STAT3, STAT5, ERα and ERK, underscoring its potential in signal transduction blockade (Fig. 4D). Moreover, N8, similar to LFA102 and N10, effectively neutralized the proliferative effects of prolactin on T47D cells (Fig. 4E). In a three-dimensional culture system, N8 effectively counteracted the antagonistic effect of PRL on tamoxifen sensitivity in T47D spheroids (Fig. 4F and Figure S5B). Given that three-dimensional tumor spheroids could better simulate in vivo conditions, this finding indicated that N8 has the potential to inhibit the effects of PRL in a physiological context. Subsequently, we generated a stable MCF7-TAMR-TETON-PRL cell line to investigate if N8 could inhibit the effect of PRL that is produced endogenously by tumor cells. The insensitivity to tamoxifen brought by overexpression of PRL in MCF7-TAMR cells could also be inhibited by N8 (Fig. 4G). Despite the comparable binding efficacy and PRL antagonizing ability of N8, N10 and LFA102 in PRLR signal blockade, N8 was particularly notable for its rapid internalization, which was an advantage for constructing immunotoxin that made us to choose it for further experiment.

### N8-PE24 immunotoxin efficiently inhibited PRLR-positive breast cancer

**Fig. 5:**
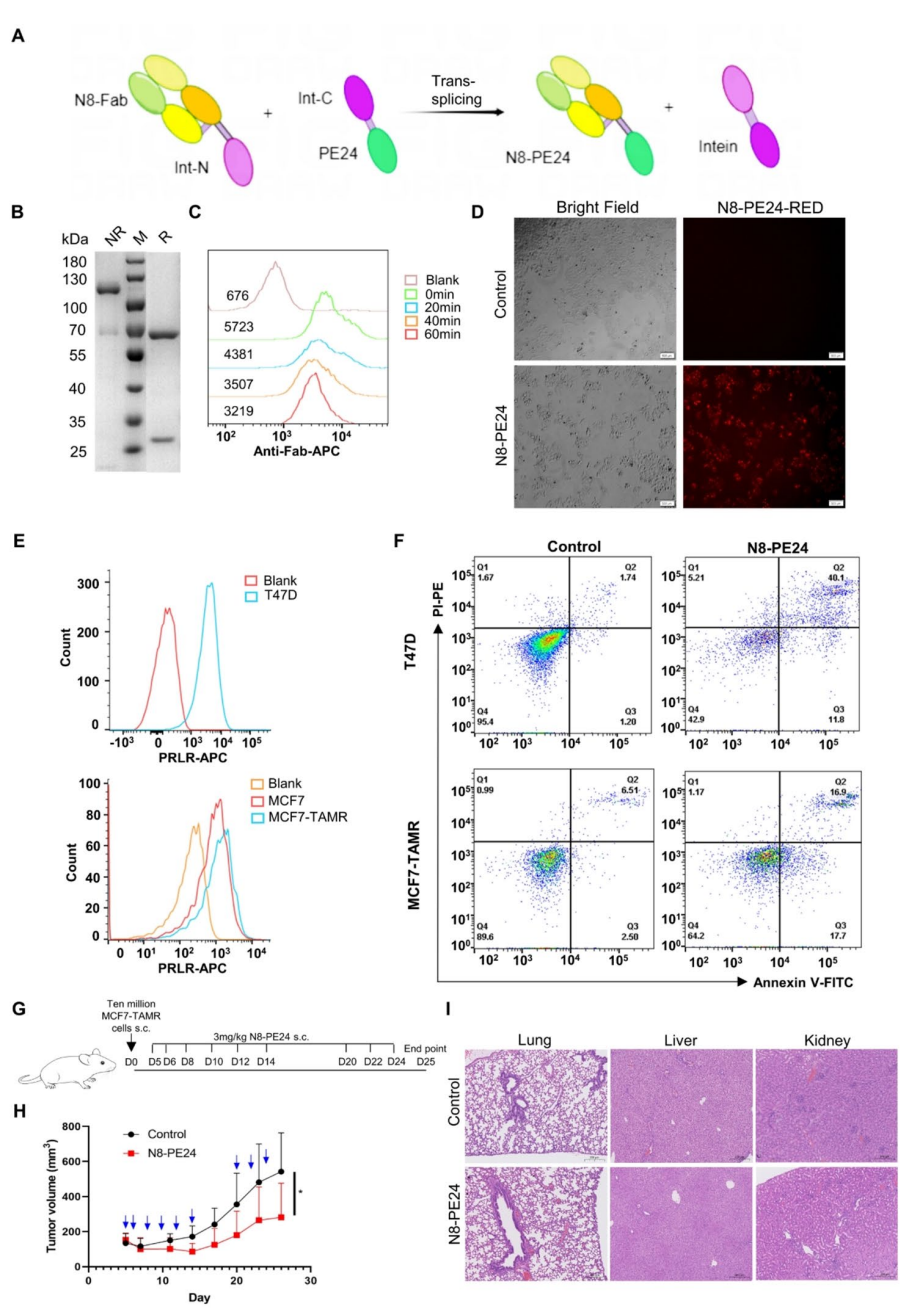
N8-PE24 immunotoxin demonstrated rapid internalization into cells and efficiently induced cell apoptosis. (**A**) Diagram of the construction of N8-PE24 immunotoxin. Int-N: N-terminal fragment of intein. Int-C: C-terminal fragment of intein. (**B**) SDS-PAGE analysis of N8-PE24. NR: non-reduced sample. R: reduced sample. (**C**) The internalization rate of N8-PE24 immunotoxin was determined by flowcytometry. After keeping the cells under 37℃ for indicated time, N8-PE24 left on cell membrane was detected by Anti-Fab-APC secondary antibody. (**D**) The internalization of pHrodo-red-labelled N8-PE24 was analyzed under fluorescence microscope. The fluorescence was visualized after culturing the cells with pHrodo-red-labelled N8-PE24 for 4 h. (**E**) PRLR level on T47D, MCF7, MCF7-TAMR cells was analyzed by flowcytometry. The PRLR was detected by anti-PRLR-APC antibody. (**F**) Apoptosis of T47D and MCF7-TAMR cells induced by N8-PE24 was analyzed by Annexin V-FITC/PI-PE staining and flowcytometry. (**G**) Dosage and treatment schedule for MCF7-TAMR xenograft model. Female SPF grade NOD/SCID mice aged 6 weeks were implanted s.c with ten million cells on day 0. When the tumor reached a volume of 100 mm^3^, treatment began. (**H**) Tumor growth curve of MCF7-TAMR xenografts treated with or without N8-PE24 on NOD/SCID mice (12 mice in each group). (**I**) HE analysis of organs from mice treated with indicated drugs

To enhance the effect of N8 as a PRLR-targeting warhead, we developed a PE24-based immunotoxin using N8, employing the splicing intein method [56, 57]. The immunotoxins, N8-PE24, were constructed by fusing PE24 toxin to the Fab fragment of N8 monoclonal antibody by splicing intein technique (Fig. 5A-B). The constructed N8-PE24 immunotoxin demonstrated rapid internalization into cells and was effective in inducing apoptosis in PRLR-positive BC cells (Fig. 5C-F). Subsequently, MCF7-TAMR cells were subcutaneously. implanted into female NOD/SCID mice to evaluate efficacy of N8-PE24 in vivo (Fig. 5G). Volume of Tumors was measured three times per week. N8-PE24 significantly inhibited MCF7-TAMR xenograft growth while did not cause loss of body weight nor toxicity to organs (Fig. 5H-I and Figure S7A).

### N8-PE24 restored the sensitivity of tamoxifen-resistant breast cancer cells to tamoxifen

**Fig. 6:**
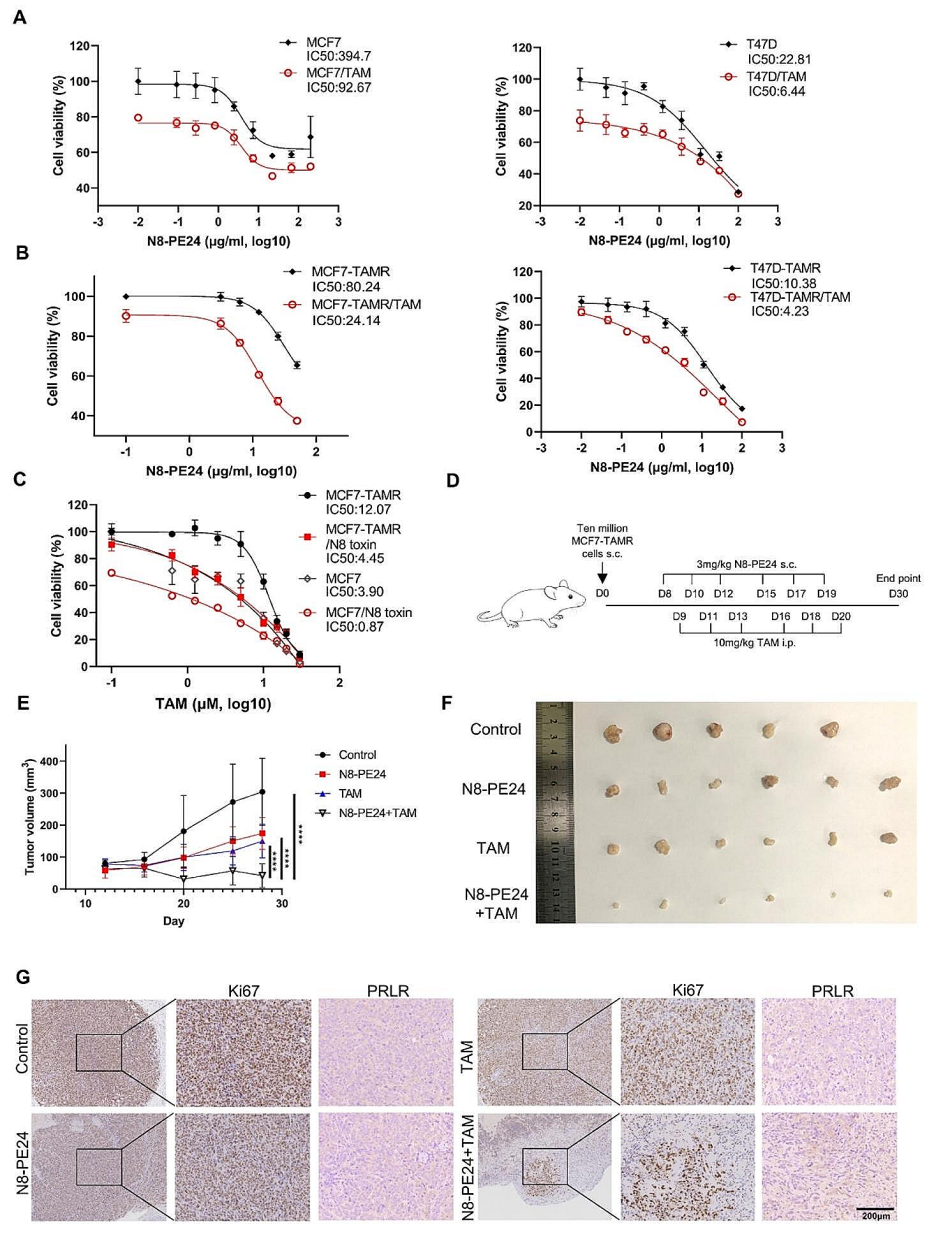
N8-PE24 combined with tamoxifen efficiently inhibited tamoxifen-resistant breast cancer cell growth. (**A**) Cell viability of MCF7 (A left) or T47D (A right) was analyzed after treatment of N8-PE24 with or without tamoxifen. Viability of cells treated without any reagents (0µM tamoxifen and 0 µg/ml N8-PE24) was set as 100%. (**B**) Cell viability of MCF7-TAMR and T47D-TAMR was analyzed after treatment of N8-PE24 with or without tamoxifen. Viability of cells treated without any reagents (0µM tamoxifen and 0 µg/ml N8-PE24) was set as 100%. (**C**) Cell viability of MCF7 or MCF7-TAMR was analyzed after treatment of serial-diluted tamoxifen with or without N8-PE24. Viability of MCF7 or MCF7-TAMR treated without any reagents (0µM tamoxifen and 0 µg/ml N8-PE24) was set as 100%. (**D**) Dosage and treatment schedule of MCF7-TAMR xenograft model treated with N8-PE24 combined with tamoxifen. Female SPF grade NOD/SCID mice aged 6 weeks were implanted s.c with ten million cells on day 0. When the tumor reached a volume of 100 mm^3^, treatment began. (**E**) Tumor growth curve of MCF7-TAMR xenografts treated with indicated drugs. (**F**) Tumor image of MCF7-TAMR xenografts treated with indicated drugs. Tumor volume was calculated as (long diameter (mm) × short diameter × short diameter (mm))/2. (**G**) IHC analysis of Ki67 and PRLR of MCF7-TAMR xenografts in different groups

Subsequently, we designed experiment to explore the synergistic effect of N8-PE24 in combination with tamoxifen. Although N8-PE24 underwent rapid intracellular degradation within cells, we observed that tamoxifen could retard this degradation process (Figure S6A). This extended degradation could be attributed to the alkalizing effect of tamoxifen, suggesting a potential synergistic interaction between tamoxifen and N8-PE24 in targeting cancer cells. Indeed, tamoxifen significantly amplified the growth-inhibitory effects of N8-PE24 on both MCF7 and T47D cells (Fig. 6A). Similar effect of tamoxifen on N8-PE24 response also occurred in MCF7-TAMR and T47D-TAMR (Fig. 6B). Moreover, the tamoxifen resistance of MCF7-TAMR was effectively overcome by the presence of N8-PE24 (Fig. 6C). We further explored the effect of the combined treatment of tamoxifen and N8-PE24 on MCF7-TAMR xenograft model (Fig. 6D). N8-PE24 combined with tamoxifen almost eradicated the tumors, demonstrating superior efficacy compared to either drug administrated alone (Fig. 6E-F). Tumors from mice treated with drug combination also exhibited reduced Ki67 expression, indicating a lower proliferative potential (Fig. 6G and Figure S7C). Concurrently, tamoxifen treatment led to an upregulation of PRLR levels in MCF7-TAMR xenografts, which is in accordance with our in vitro results (Fig. 6G and Figure S7D). Additionally, the drug combination caused no obvious loss of body weight (Figure S7B). Collectively, these results suggested that the combination of N8-PE24 and tamoxifen was effective at inhibiting BC cell growth.

### N8-PE24 immunotoxin enhanced sensitivity of chemotherapy in PRLR-positive triple-negative breast cancer xenograft

**Fig. 7:**
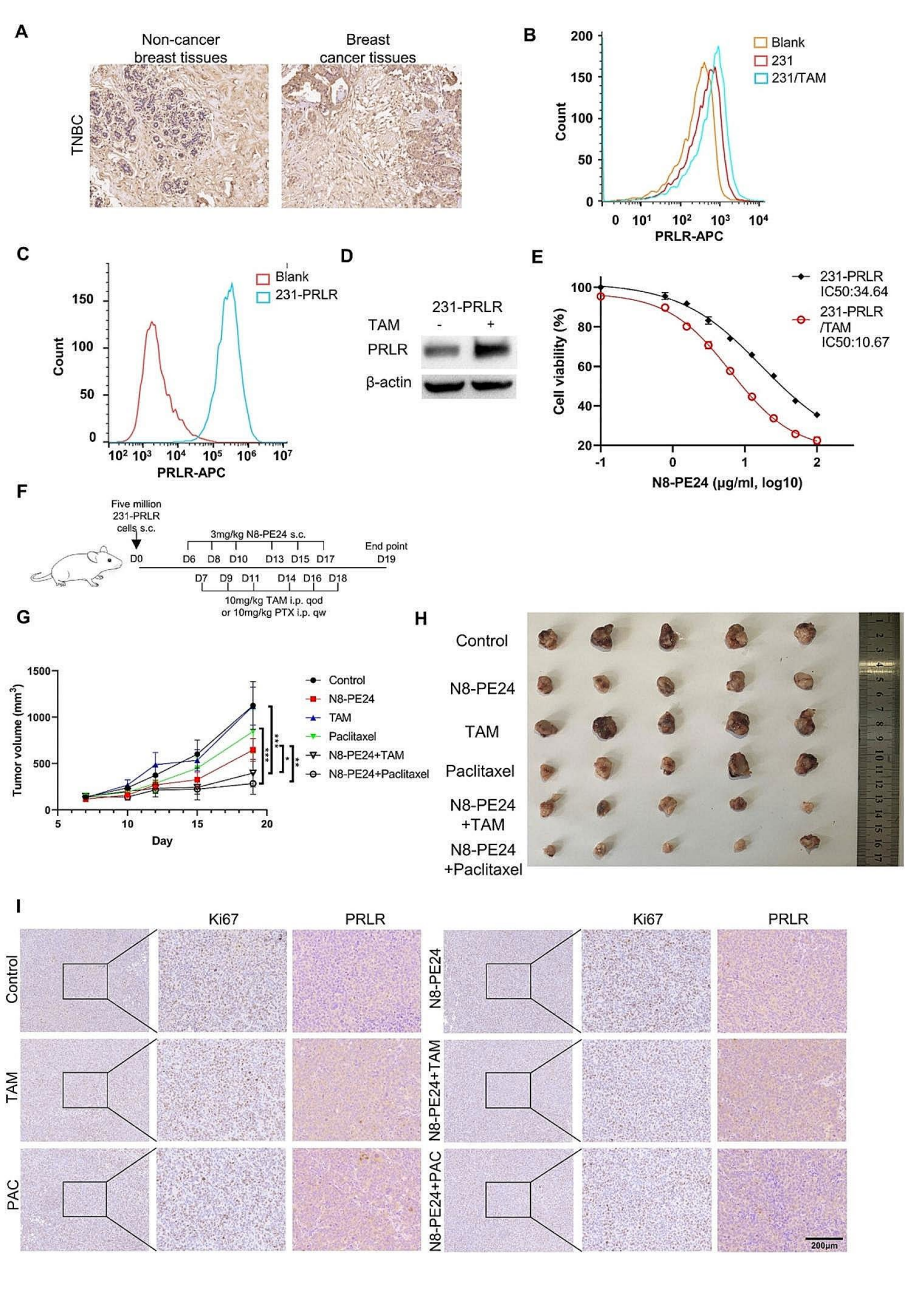
N8-PE24 combined with tamoxifen or paclitaxel could inhibit 231-PRLR breast cancer xenograft. (**A**) PRLR IHC analysis of tumor and adjacent normal tissues from a TNBC patient. (**B**) Flowcytometry analysis of PRLR on MDA-MB-231 cells treated with or without tamoxifen. 2.5µM tamoxifen was added to cells for 2 days before analysis. Anti-PRLR-APC antibody was used for staining. (**C**) Flowcytometry analysis of PRLR on 231-PRLR cells. Isotype-APC (red) and anti-PRLR-APC antibody (blue) were used for staining. (**D**) Western blot determining PRLR protein level in 231-PRLR cells when tamoxifen was present or not. 2.5µM tamoxifen was added to cells for 2 days before cell were collected and lysed. Cell lysates were then probed by anti-PRLR antibody, (**E**) Evaluation of cell viability by CCK8 assay to determine inhibition effect of N8-PE24 when tamoxifen was present or not on 231-PRLR BC. Viability of 231-PRLR cells treated without any reagents (0µM tamoxifen and 0 µg/ml N8-PE24) was set as 100%. (**F**) Dosage and treatment schedule for 231-PRLR xenograft model. Female SPF grade Balb/c nude mice aged 6 weeks were implanted s.c with five million cells on day 0. When the tumor reached a volume of 100 mm^3^, treatment began. (**G**) Tumor growth curve of 231-PRLR xenografts treated with indicated drugs on nude mice. Tumor volume was calculated as (long diameter (mm) × short diameter (mm) × short diameter (mm))/2. (**H**) Tumor image of 231-PRLR xenografts treated with indicated drugs. (**I**) IHC analysis of Ki67 and PRLR of 231-PRLR xenografts in different groups

PRLR could be highly expressed in some cases of triple-negative breast cancer (TNBC) (Fig. 7A and Figure S1D). Thus, it’s necessary to evaluate N8-PE24 efficacy in PRLR-positive TNBC. To investigate the potential of tamoxifen to elevate PRLR level in TNBC cells, we treated MDA-MB-231 cells with tamoxifen. Our results indicated that tamoxifen treatment induced a modest upregulation of membrane-bound PRLR on MDA-MB-231 cells (Fig. 7B). However, the basal PRLR level in MDA-MB-231 was too low to warrant further investigation. Thus, we constructed MDA-MB-231 cells expressing PRLR (231-PRLR) for further experiment (Fig. 7C). As expected, tamoxifen treatment significantly upregulated PRLR level in 231-PRLR (Fig. 7D). Remarkably, 231-PRLR cells demonstrated an augmented response to N8-PE24 in the presence of tamoxifen (Fig. 7E). This suggested that tamoxifen could enhance the therapeutic efficacy of N8-PE24 even in estrogen receptor-negative, yet PRLR-positive, breast tumor cells. Encouraged by these findings, we extended our investigation in vivo using 231-PRLR xenografts. Five million 231-PRLR breast cancer cells were s.c. implanted on female nude mice and treatment initiated when the tumors reached a volume of approximately 100mm^3^ (Fig. 7F). Tamoxifen and paclitaxel were used as negative and positive controls, respectively. Despite tamoxifen alone showed no inhibitory effect on 231-PRLR xenografts, the therapeutic efficacy was markedly elevated when N8-PE24 was co-administrated with tamoxifen (Fig. 7G-H). Furthermore, co-administration of N8-PE24 with paclitaxel also demonstrated a remarkable tumor-suppressing effect (Fig. 7G-H). Paclitaxel exerted inhibitory functions through disrupting mitosis, a mechanism different with PE24 [63]. Thus, the synergistic efficacy of N8-PE24 in combination with paclitaxel suggested that N8-PE24 could be administrated with other cytotoxic drugs to provide further benefits. Notably, tumors subjected to combination therapy with N8-PE24 and either tamoxifen or paclitaxel exhibited a lower percent of Ki67 area, indicative of a lower proliferative potential, compared to those treated with monotherapy (Fig. 7I). Meanwhile, tamoxifen treatment was observed to elevate PRLR level within xenografts, potentially accounting for the synergistic effect when N8-PE24 was combined with tamoxifen (Fig. 7I and Figure S7G). In summary, the combination of N8-PE24 with tamoxifen or paclitaxel efficiently inhibited tumor growth in 231-PRLR xenografts model, highlighting the potential broad-spectrum applicability of this therapeutic strategy on BC.

## Discussions

Tamoxifen, which competes with estradiol for binding AF-2 domain, is one of the standard anti-breast cancer drugs used in clinical practice [64]. However, the resistance to tamoxifen is a serious clinical challenge. Activation of MAPK/ERK pathways could result in ligand-independent ERα activation, leading to subsequent tamoxifen resistance [41, 65, 66]. Previous study revealed that PD98059, a MEK inhibitor, efficiently inhibits PRL-induced ERα phosphorylation [14]. In our study, we identified that PRL induced phosphorylation of ERα by ERK, a process that could be inhibited by SCH72984, a specific ERK inhibitor. In dense collagen matrices, supplement of PRL mitigates the sensitivity of BC cells to tamoxifen [67]. It indicates that PRL might affect the efficacy of tamoxifen treatment in solid tumor. Indeed, plasma PRL was correlated with BC progression after tamoxifen treatment [68]. Our data further showed that PRL could decrease tamoxifen sensitivity of breast cancer in a 3D spheroids model. It has been demonstrated that PRL is also a local BC promoter that predicts bad prognosis [69]. Here, we found that tumor PRL level predicted shorter RFS in patients treated with tamoxifen. We identified that the overexpression of PRL in BC cells desensitized the cells to tamoxifen. However, the mechanisms by which tumors could regulate local PRL expression still remain to be further illustrated.

A selection of preexisting epigenetically distinct cells could finally cause the occurrence of tamoxifen-resistant cells [70]. We found that in multiple datasets, the tamoxifen-resistant BC cells exhibit an upregulation of PRLR level. Although a modest increase of PRLR transcription was observed in several BC cells following short-term tamoxifen exposure, a significant and profound upregulation of PRLR transcription was noted in MCF7-TAMR cells screened by long-term tamoxifen treatment. This could be the result of selection by tamoxifen pressure. Tamoxifen could also mediate lysosome alkalization independent of ERα [71, 72]. As an alkalizing agent, tamoxifen could upregulate pH in lysosome and increase lysosome permeability [60–62]. Physiologically, PRLR is constitutively trafficked into lysosomes, where it was rapidly degraded [58, 59]. In our study, we identified the alkalizing effect of tamoxifen on lysosomes and its subsequent degradation of PRLR. Interestingly, short-term tamoxifen treatment could significantly upregulated PRLR at protein level, potentially due to the role of tamoxifen as a lysosome alkalizing agent. Our data further indicates that tamoxifen could be combined with PRLR-targeting therapy.

**Fig. 8:**
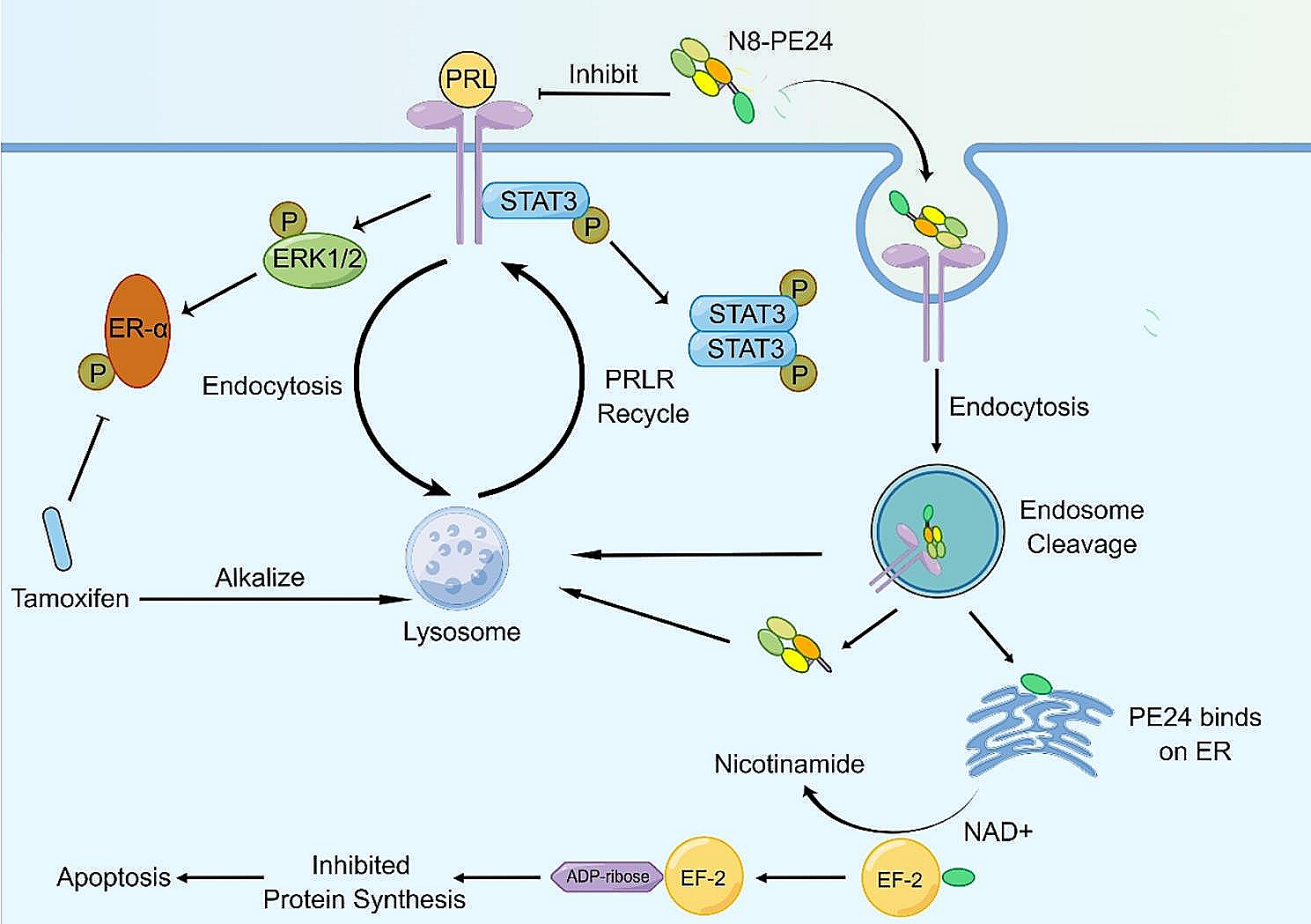
Concepts of employing N8-PE24 combined with tamoxifen to inhibit breast cancer cells. PRL promotes phosphorylation of ERα, which could induce resistance to tamoxifen. N8-PE24 could restore sensitivity of breast cancer cells to tamoxifen. Meanwhile, tamoxifen could induce accumulation of PRLR in breast cancer cells through alkalizing lysosomes, thereby promoting the effect of N8-PE24. After being internalized into cells, PE24 is released from N8-PE24 and transfers ADP-ribose on EF-2, inducing apoptosis through inhibiting protein synthesis. The picture was drawn by FigDraw

Tumor progression attributes to comprehensive factors. Although the activation of PRLR pathway has been identified as a cancer promoter, targeting PRL-PRLR axis alone achieves limited effects in clinical trials [26, 27]. Given the extensive crosstalk between PRL-PRLR axis and other pathways, including ERα and IGF-1R, the combination of PRLR-targeting therapy with other targeted drugs might achieve superior effect [31]. The development of ADC represents an alternative strategy to overcome drug insensitivity caused by compensatory signaling pathways. For instance, T-DM1 and T-Dxd are ADCs that combine the anti-tumoural effect of cytotoxics and Herceptin into a single pharmacological entity, demonstrating greater efficacy than the sum of their individual parts [73]. An ADC targeting PRLR, termed ABBV-176, could deliver pyrrolobenzodiazepine into cancer cells and significantly inhibits BC cells preclinically [44]. However, ABBV-176 has been associated with cumulative toxicity in patients [45]. Immunotoxin could offer a safer alternative due to the absence of payload dissociation [46]. Here, we designed and evaluated the efficacy of N8-PE24, an immunotoxin that was constructed by intein methods and targets PRLR [56, 57, 74]. N8-PE24 could inhibit BC cells through two mechanisms. First, N8 mAb could efficiently inhibit signals downstream PRLR and restore tamoxifen sensitivity in BC cells. Second, the rapid internalization of N8-PE24 could deliver PE24 part into cells. Upon binding with KDEL receptor in the endoplasmic reticulum, the PE24 fragment could transfer ADP-ribose to elongation factor 2 (EF2), inhibiting protein synthesis and inducing cell apoptosis [47, 48]. Interestingly, tamoxifen could prolong retention of N8-PE24 within cells. And we found that tamoxifen could promote N8-PE24 effect in multiple BC cell lines, including T47D-TAMR, MCF7-TAMR and 231-PRLR. The synergistic effects of tamoxifen and N8-PE24 could be attributed to PRLR upregulation and N8-PE24 retention. Indeed, other alkalizing agent like ammonium chloride has been utilized to enhance the effects of immunotoxin [75]. Similarly, tamoxifen has been employed to facilitate the release of docetaxel from lysosomes [76]. In our study, we identified the efficacy of tamoxifen combined with immunotoxin in xenograft models. The results observed in 231-PRLR models indicate that tamoxifen could be used as alkalizing agent to promote effects of immunotoxin. Furthermore, as previously reported, PRLR antagonism could reduce the clonogenic capacity of BC cells and potentiate cytotoxicity of paclitaxel [29]. We confirmed the efficacy of N8-PE24 combined with paclitaxel in mice bearing 231-PRLR xenografts. In summary, our findings could provide a new perspective for employing tamoxifen combined with other cytotoxic drugs (Fig. 8).

## Conclusions

Our study preliminarily found that tamoxifen could upregulate PRLR level in breast cancer cells. Besides, activation of PRLR pathway by PRL could desensitize breast cancer cells to tamoxifen. Based on these, we design N8-PE24 immunotoxin and identify its efficacy in restoring drug sensitivity to tamoxifen both in vitro and in vivo. What’s more, N8-PE24 significantly improve the efficacy of chemotherapy in PRLR-positive TNBC or xenograft models. Our study provides a new perspective for targeting PRLR to treat breast cancer.

### Electronic supplementary material

Below is the link to the electronic supplementary material.

Supplementary Material 1

## Data Availability

All data generated or analyzed are included in this article or available online.

## Abbreviations

PRL: Prolactin
PRLR: Prolactin receptor
ERα: Estrogen receptor α
RFS: Relapse free survival
BC: Breast cancer
TNBC: Triple-negative breast cancer
FBS: Fetal bovine serum
BSA: Bovine serum albumin
PBS: Phosphate buffer saline
DMEM: Dulbecco’s modified Eagle’s medium
mAb: Monoclonal antibody
ADC: Antibody-drug conjugate
CHX: Cycloheximide
TAM: Tamoxifen
BAF: Baflomycin A1
